# Current use of inotropes in circulatory shock

**DOI:** 10.1186/s13613-021-00806-8

**Published:** 2021-01-29

**Authors:** Thomas W. L. Scheeren, Jan Bakker, Thomas Kaufmann, Djillali Annane, Pierre Asfar, E. Christiaan Boerma, Maurizio Cecconi, Michelle S. Chew, Bernard Cholley, Maria Cronhjort, Daniel De Backer, Arnaldo Dubin, Martin W. Dünser, Jacques Duranteau, Anthony C. Gordon, Ludhmila A. Hajjar, Olfa Hamzaoui, Glenn Hernandez, Vanina Kanoore Edul, Geert Koster, Giovanni Landoni, Marc Leone, Bruno Levy, Claude Martin, Alexandre Mebazaa, Xavier Monnet, Andrea Morelli, Didier Payen, Rupert M. Pearse, Michael R. Pinsky, Peter Radermacher, Daniel A. Reuter, Yasser Sakr, Michael Sander, Bernd Saugel, Mervyn Singer, Pierre Squara, Antoine Vieillard-Baron, Philippe Vignon, Jean-Louis Vincent, Iwan C. C. van der Horst, Simon T. Vistisen, Jean-Louis Teboul

**Affiliations:** 1 Department of Anesthesiology, University of Groningen, University Medical Center Groningen, Hanzeplein 1, P.O.Box 30.001, 9700 RB Groningen, The Netherlands; 2 New York University Medical Center, New York, USA; 3 Columbia University Medical Center, New York, USA; 4 Erasmus MC University Medical Center Rotterdam, Rotterdam, The Netherlands; 5 Pontificia Universidad Católica de Chile, Santiago, Chile; 6 School of Medicine Simone Veil, Raymond Poincaré Hospital (APHP), Department of Intensive Care Medicine, University of Versailles- University Paris Saclay, Garches, France; 7 Département de Médecine Intensive-Réanimation Et de Médecine Hyperbare, Centre Hospitalier Universitaire Angers; and Institut MITOVASC, CNRS UMR 6215, INSERM U1083, Angers University, Angers, France; 8 Medical Centre Leeuwarden, Department of Intensive Care, Leeuwarden, the Netherlands; 9 Department of Anesthesia and Intensive Care, IRCCS Humanitas Research Hospital, Via Manzoni 56, Milan, Italy; 10 Department of Biomedical Sciences, Humanitas University, Via Rita Levi Montalcini, Milan, Italy; 11 Department of Anaesthesiology and Intensive Care, Biomedical and Clinical Sciences, Linköping University, Linköping, Sweden; 12 Department of Anaesthesiology & Intensive Care Medicine, AP-HP, Hôpital Européen Georges Pompidou, Paris, France; 13 Université de Paris, Paris, France; 14 Section of Anaesthesiology and Intensive Care, Department of Clinical Science and Education, Karolinska Institutet, Södersjukhuset, Stockholm, Sweden; 15 Department of Intensive Care, CHIREC Hospitals, Université Libre de Bruxelles, Brussels, Belgium; 16 Cátedra de Farmacología Aplicada, Facultad de Ciencias Médicas, Universidad Nacional de La Plata Y Servicio de Terapia Intensiva, Sanatorio Otamendi, Buenos Aires, Argentina; 17 Department of Anesthesiology and Intensive Care Medicine, Kepler University Hospital and Johannes Kepler University Linz, Linz, Austria; 18 Department of Anaesthesia and Intensive Care, Assistance Publique Des Hopitaux de Paris, Hôpitaux Universitaires Paris-Saclay, Université Paris-Saclay, Hôpital de Bicêtre, Le Kremlin-Bicêtre, France; 19 Division of Anaesthetics, Pain Medicine and Intensive Care, Imperial College London, London, UK; 20 Department of Cardiopneumology, Instituto Do Coracao, Universidade de São Paulo, Hospital SirioLibanes, São Paulo, Brazil; 21 Assistance Publique-Hôpitaux de Paris, Paris Saclay University Hospitals, Antoine Béclère Hospital, Paris, France; 22 Departamento de Medicina Intensiva, Facultad de Medicina, Pontificia Universidad Católica de Chile, Santiago, Chile; 23 Servicio de Terapia Intensiva, Hospital Fernández, Buenos Aires, Argentina; 24 Department of Critical Care, University of Groningen, University Medical Center Groningen, Groningen, the Netherlands; 25 Department of Anesthesia and Intensive Care, IRCCS San Raffaele Scientific Institute, Vita-Salute San Raffaele University, Milan, Italy; 26 Aix Marseille Université, Assistance Publique Hôpitaux de Marseille, Service D’Anesthésie Et de Réanimation CHU Nord, Marseille, France; 27 Service de Réanimation Médicale Brabois Et Pôle Cardio-Médico-Chirurgical. CHRU Brabois, INSERM U1116, Université de Lorraine, Vandoeuvre les NancyNancy, 54500 France; 28 Department of Anesthesia, Burn and Critical Care, APHP Hôpitaux Universitaires Saint Louis LariboisièreUniversité Paris DiderotU942 Inserm, Paris, France; 29 Medical Intensive Care Unit, Assistance Publique-Hôpitaux de Paris, Paris-Saclay University Hospitals, Bicêtre hospital, Le Kremlin-Bicêtre, France; 30 INSERM UMR_S 999, FHU SEPSIS, Le Kremlin-Bicêtre, France; 31 Department of Clinical Internal, Anesthesiological and Cardiovascular Science, Sapienza University of Rome, Rome, Italy; 32 University Paris 7 Denis Diderot; INSERM 1160 and Hôpital Lariboisière, APHP, Paris, France; 33 William Harvey Research Institute, Queen Mary University of London, London, EC1M 6BQ UK; 34 Department of Critical Care Medicine, University of Pittsburgh, Pittsburgh, USA; 35 Institut Für Anästhesiologische Pathophysiologie Und Verfahrensentwicklung, Universitätsklinikum Ulm, Ulm, Germany; 36 Department of Anesthesiology and Intensive Care Medicine, Rostock University Medical Centre, Rostock, Germany; 37 Department of Anesthesiology and Intensive Care, Uniklinikum Jena, Jena, Germany; 38 Department of Anesthesiology, Intensive Care Medicine and Pain Therapy, University Hospital Giessen, UKGM, Justus-Liebig University Giessen, Giessen, Germany; 39 Department of Anesthesiology, Center of Anesthesiology and Intensive Care Medicine, University Medical Center Hamburg-Eppendorf, Hamburg, Germany; 40 Bloomsbury Institute of Intensive Care Medicine, Division of Medicine, University College London, London, UK; 41 ICU Department, Réanimation CERIC, Clinique Ambroise Paré, Neuilly, France; 42 Assistance Publique-Hôpitaux de Paris, University Hospital Ambroise Paré, intensive care unit, Boulogne-Billancourt, France; 43 INSERM U-1018, CESP, Team 5, University of Versailles Saint-Quentin en Yvelines, Villejuif, France; 44 Medical-Surgical Intensive Care Unit, INSERM CIC-1435, Teaching Hospital of Limoges, Limoges, France; 45 University of Limoges, Limoges, France; 46 Université Libre de Bruxelles - Dept of Intensive Care, Erasme Univ Hospital, Brussels, Belgium; 47 Department of Intensive Care Medicine, Maastricht University Medical Center, Maastricht, The Netherlands; 48 Institute of Clinical Medicine, Aarhus University, Aarhus, Denmark; 49 Department of Anesthesia and Intensive Care, Aarhus University Hospital, Aarhus, Denmark

**Keywords:** Acute circulatory failure, Sepsis, Septic shock, Cardiogenic shock, Resuscitation, Inotropes, Vasoactive agents, Catecholamines, Levosimendan, PDE-inhibitors, Cardiac output

## Abstract

**Background:**

Treatment decisions on critically ill patients with circulatory shock lack consensus. In an international survey, we aimed to evaluate the indications, current practice, and therapeutic goals of inotrope therapy in the treatment of patients with circulatory shock.

**Methods:**

From November 2016 to April 2017, an anonymous web-based survey on the use of cardiovascular drugs was accessible to members of the European Society of Intensive Care Medicine (ESICM). A total of 14 questions focused on the profile of respondents, the triggering factors, first-line choice, dosing, timing, targets, additional treatment strategy, and suggested effect of inotropes. In addition, a group of 42 international ESICM experts was asked to formulate recommendations for the use of inotropes based on 11 questions.

**Results:**

A total of 839 physicians from 82 countries responded. Dobutamine was the first-line inotrope in critically ill patients with acute heart failure for 84% of respondents. Two-thirds of respondents (66%) stated to use inotropes when there were persistent clinical signs of hypoperfusion or persistent hyperlactatemia despite a supposed adequate use of fluids and vasopressors, with (44%) or without (22%) the context of low left ventricular ejection fraction. Nearly half (44%) of respondents stated an adequate cardiac output as target for inotropic treatment. The experts agreed on 11 strong recommendations, all of which were based on excellent (> 90%) or good (81–90%) agreement. Recommendations include the indications for inotropes (septic and cardiogenic shock), the choice of drugs (dobutamine, not dopamine), the triggers (low cardiac output and clinical signs of hypoperfusion) and targets (adequate cardiac output) and stopping criteria (adverse effects and clinical improvement).

**Conclusion:**

Inotrope use in critically ill patients is quite heterogeneous as self-reported by individual caregivers. Eleven strong recommendations on the indications, choice, triggers and targets for the use of inotropes are given by international experts. Future studies should focus on consistent indications for inotrope use and implementation into a guideline for circulatory shock that encompasses individualized targets and outcomes.

## Background

Circulatory shock affects about one-third of patients admitted to the intensive care unit (ICU) [1]. Shock is defined as insufficient oxygen and energy supply to organs and is associated with increased mortality [1, 2]. Traditionally, four types of circulatory shock have been distinguished by pathophysiological mechanisms, namely hypovolemic, cardiogenic, distributive and obstructive shock [3]. Critically ill patients present with one or a combination of these four types of circulatory failure [4].

Treatment of circulatory shock relies on timely initiation of adequate fluid resuscitation combined with the use of vasoactive medication to restore tissue perfusion [5, 6]. Despite these therapeutic measures, cardiac output (CO) is often inadequate to deliver enough oxygen to tissues in patients with circulatory shock [7]. Inotropes might improve CO and organ perfusion in patients with circulatory shock [8, 9]. Several guidelines for different types of circulatory shock give different recommendations for the use of inotropes [10–13]. Despite these different recommendations and the apparent lack of evidence, inotropes are used in daily practice [13, 14]. Data on how inotropes are used in clinical practice are sparse [15]. Individual registries, observational studies, and trials with patients in shock provide insight into the current standard of care. For example, in patients with cardiogenic shock, vasopressors and inotropes were administered in 94%, where dobutamine (49%) and levosimendan (24%) were the most commonly used inotropes [16]. For levosimendan, two systematic reviews with meta-analyses and three large randomized trials have shown neutral effects on various outcomes [17–21], while one trial reported a possibility of harm (lower likelihood of successful weaning from mechanical ventilation and a higher risk of supraventricular tachyarrhythmia)[22]. A recent Cochrane review underlines the low quality of evidence on the use of inotropes in cardiogenic shock with levosimendan showing a short-term survival benefit over dobutamine, while this benefit vanished on long-term follow-up [23]. In other types of shock, use of inotropes is less common. Some patients with septic shock may have improved tissue perfusion with inotropic therapy aimed at increasing oxygen delivery and in this situation, dobutamine is the first-line inotrope [8, 24]. However, a recent network meta-analysis suggests that levosimendan has the highest probability of being the best treatment in septic shock [25]. Yet, no large randomized trials have provided evidence for a mortality benefit of levosimendan over dobutamine in septic shock [26].

Hence, further studies are needed on optimal treatment with inotropes in circulatory shock states. To aid the design and interpretation of future studies on inotropes, it is imperative to evaluate current practice and therapeutic goals of inotropic treatment of shock states to establish what is considered *standard of care*. The aim of the present study was to establish an overall picture of the standard of care, which was identified from a survey among members of the European Society of Intensive Care Medicine (ESICM). Furthermore, we developed recommendations on the use of inotropes based on a subsequent questionnaire and consensus finding by international experts in the field.

## Methods

### Survey development

Survey questions and response options were developed by the leadership of the Cardiovascular Dynamics Section of ESICM. The survey consisted of 27 questions on the use of vasoactive drugs. The first results on the current use of vasopressors in septic shock were recently published [6]. The present study focused on 14 survey questions related to the use of inotropes in circulatory shock. These questions concerned triggering factors, first-line drug choice, dosing, timing, targets, additional treatment strategies, and effects of inotropes.

The Research Committee of the ESICM endorsed the survey. Data were collected automatically using SurveyMonkey Inc. (www.surveymonkey.com).

The survey was announced on the ESICM website and was open for participation between November 2016 and April 2017. Members of the Cardiovascular Dynamics section of the ESICM were additionally encouraged to participate via an email linking to the survey sent to email addresses in ESICM’s membership database in November 2016 with two subsequent e-mail reminders in February 2017 and March 2017. No incentives were offered for participation. No personal information was collected, and no log-in was required to participate. Completing the internal consistency of items was enforced by displaying an alert before the questionnaire could be submitted and highlighting mandatory but unanswered questions. It was not possible to review and change the given answers after submission.

### Survey reporting

The questionnaire's methodology and results are reported according to the Checklist for Reporting Results of Internet E-Surveys (CHERRIES) statement [27]. Ethical approval for this study was not requested, as no identifying data were collected and consent was assumed by participating in the survey.

### Experts’ recommendations

Based on the results of the ESICM members’ survey, three authors (TWLS, IVDH and JLT) identified areas of interest and developed 11 questions, including sub-questions and approached a group of 42 experts, who are active members of the Cardiovascular Dynamics (CD) section of the ESICM. These experts have all published research as first or last author in an international peer-reviewed journal in articles identified by the PubMed subject headings “inotrope”, and they were asked to answer the developed *experts’ questionnaire* in order to summarize overall recommendations for the use of inotropes in circulatory shock based on the ESICM members’ survey and the experts’ questionnaire.

Definitions of the degree of consensus and grades of recommendations were based on the RAND algorithm [28]. Excellent agreement (> 90% agreement) and good agreement (81–90% agreement) were considered as strong grades of recommendation. A weak agreement was defined when 70–80% of the experts agreed. A Delphi-like process was used to achieve these consensus grades.

### Statistics

Data were evaluated as the total distribution of single answers. Answers to the questionnaire items are reported as numbers (percentage). Contingency tables and corresponding Chi-square statistics were reported to describe the pairwise associations between selected demographic variables (European vs. non-European ESICM member, high-income vs. lower-income countries, ICU experience more vs. less than 5 years full time, intensive care as primary specialty vs. other specialties, and university hospital vs. non-university hospital) and the responses regarding inotrope use. The nature of each question’s five answer options and their distribution prospectively defined the answer categories for the subsequent contingency tables analyses (2 × 2). In two cases, only three (question 3) and two (question 7) answer options were used two define the two answer categories for contingency tables. We used the World Bank definition of a “high-income country,” i.e., a per capita gross national income of $12,056 or more [29]. *P* < 0.05 was considered statistically significant. P-values are reported with their exact value for interpretation and not corrected for multiple testing in this descriptive reporting.

## Results

### Survey respondents’ characteristics

A total of 839 physicians from 82 countries participated in the survey. A firm estimate of response rate could not be calculated as the invitation to the survey was posted as an open link on the ESICM website. In addition, members of the CD section of the ESICM (*n* = 10,780 at the time of the survey) received an email invitation to participate. From these addressees, 3111 (29%) opened this e-mail (according to Mail Chimp). This corresponds to a response rate of 27% (839/3111) of those who opened the e-mail. Baseline demographic data of respondents and their ICU and hospitals are presented in Additional file 1: Table S1 [6].

### Survey results

All seven questions and answers of the respondents on inotrope use in circulatory shock are summarized in Table 1. Dobutamine was reported to be used as the first-line inotrope in 704 (84%) of questionnaire respondents, followed by PDE-inhibitors (7%), levosimendan (5%), dopamine (4%), and dopexamine (0.1%), while epinephrine was not among the first 5 most used agents. First-line use of dobutamine was more common among non-Europeans than Europeans (88% vs. 82%, *p* = 0.049).

**Table 1:** Survey questions on inotropic use

	Frequency of response	Percentage of response
What is your first-line inotrope to increase cardiac pump function?		
Dobutamine	706	84%
Dopamine	37	4%
Dopexamine	1	0.1%
Levosimendan	38	5%
Milrinone or any other phosphodiesterase inhibitor	57	7%
What are your most important criteria for using an inotrope to increase cardiac pump function?		
I measure CO and find it low (e.g., cardiac index < 2.5 L min^−1^ m^−2^)	189	23%
I measure central venous oxygen saturation and find it low (< 70%)	47	6%
I measure left ventricular ejection fraction and find it low (< 45%)	54	6%
There are persistent clinical signs of hypoperfusion (e.g., skin mottling, low urine output) or persistent hyperlactatemia despite a supposed adequate use of fluids and vasopressors	184	22%
There are persistent clinical signs of hypoperfusion (e.g., skin mottling, low urine output) or persistent hyperlactatemia despite a supposed adequate use of fluids and vasopressors in the context of low left ventricular ejection fraction	365	44%
What are your primary therapeutic targets when using an inotrope?		
A normal lactate level	155	18%
A normal veno-arterial PCO_2_ difference (< 6 mmHg)	37	4%
An adequate CO	372	44%
An adequate ScvO_2_	173	21%
An adequate urine output	102	12%
When the patient does not respond to your current inotropic therapy, what is your main reason for adding another inotrope/vasoactive agent to the current therapy?		
Although the maximum dose of the 1st choice inotrope has not been reached, previous increases in the inotrope dose were ineffective	129	15%
By adding a second inotrope although the maximum dose of the 1st choice inotrope has not been reached, I want to limit/reduce the side effects of the first inotrope	193	23%
I suppose that the mechanism of action of the first inotrope is exhausted (e.g., adrenoceptors down regulation) and want to use a second one with an independent mechanism of action	195	23%
I want to use synergistic effects of two different mechanisms of action	233	28%
The maximum dose of the 1st choice inotrope has been reached	89	11%
Which of the following statements fits best your opinion on catecholamine use in the treatment of shock?		
Although epinephrine (EPI) has the same adrenoceptors profile than the combination norepinephrine (NOR) plus dobutamine (DOB), the combination NOR plus DOB should be preferred since either component can be titrated individually	372	44%
Although EPI has the same adrenoceptors profile than the combination NOR plus DOB, EPI should be preferred because of its easiness to be used (single agent)	50	6%
The combination NOR plus DOB should be preferred over EPI due to a better patient outcome	95	11%
The combination NOR plus DOB should be preferred over EPI since EPI may decrease regional blood flow, particularly in the splanchnic circulation	131	16%
The combination NOR plus DOB should be preferred over EPI since EPI may increase blood lactate levels and cause cardiac arrhythmias	191	23%
Which of the following statements fit(s) best your opinion on the use of phosphodiesterase (PDE)-inhibitors in the critically ill?		
Because of their prominent vasodilatory effect on the pulmonary circulation, PDE-inhibitors should be preferred in the treatment of predominant right heart failure	365	43%
Compared to pure vasodilators (e.g., nitroprusside), PDE-inhibitors cause larger increases in CO and smaller decreases in arterial pressure	191	23%
Compared to ß-adrenoceptor agonists (e.g., dobutamine), PDE-inhibitors have similar effects on CO but additionally decrease the cardiac filling pressures (CVP, PAOP)	162	19%
PDE-inhibitors should be avoided in the treatment of cardiogenic shock since they are associated with increased mortality	74	9%
PDE-inhibitors should be avoided in the treatment of cardiogenic shock since they may increase the incidence of atrial fibrillation or tachyarrhythmias	47	6%
Which of the following statements fits best your opinion on the use of levosimendan in the critically ill?		
Levosimendan is the only inotrope that does not increase myocardial oxygen demand	354	42%
Levosimendan is also a potent vasodilator in the systemic and pulmonary circulation	269	32%
Levosimendan is associated with an increased incidence of atrial fibrillation and ventricular ectopy	83	10%
Levosimendan is the only inotrope that is not associated with an increased mortality	55	7%
Levosimendan may be considered as cardioprotective, as it reduces troponin I release (pleiotropic effect)	78	9%

According to respondents, most inotropes are used when *there are persistent clinical signs of hypoperfusion (e.g., skin mottling, low urine output) or persistent hyperlactatemia despite a supposed adequate use of fluids and vasopressors* (65%) (Table 1).

Mostly, *an adequate CO* was the preferred *target for inotropic treatment* (44%) (Table 1).

The reasons for adding another inotrope when the patient did not respond to the first-line inotropic therapy varied among respondents (Table 1).

Most respondents preferred the *combination of norepinephrine plus dobutamine over epinephrine as preferred catecholamine* in the treatment of circulatory shock (Table 1).

Concerning the use of phosphodiesterase (PDE)-inhibitors, respondents employed by a university hospital and more experienced respondents were more likely to support PDE-inhibitors in right heart failure than non-university or less experienced respondents (52% vs. 37%, *p* < 0.001 and 48% vs. 39%, *p* = 0.01, respectively) (Table 1).

Responses to the use of levosimendan varied among respondents, with experienced clinicians more likely selecting levosimendan than less experienced clinicians (61% vs. 52%, *p* = 0.01) (Table 1).

### Experts’ questionnaire results

**Fig. 1:**
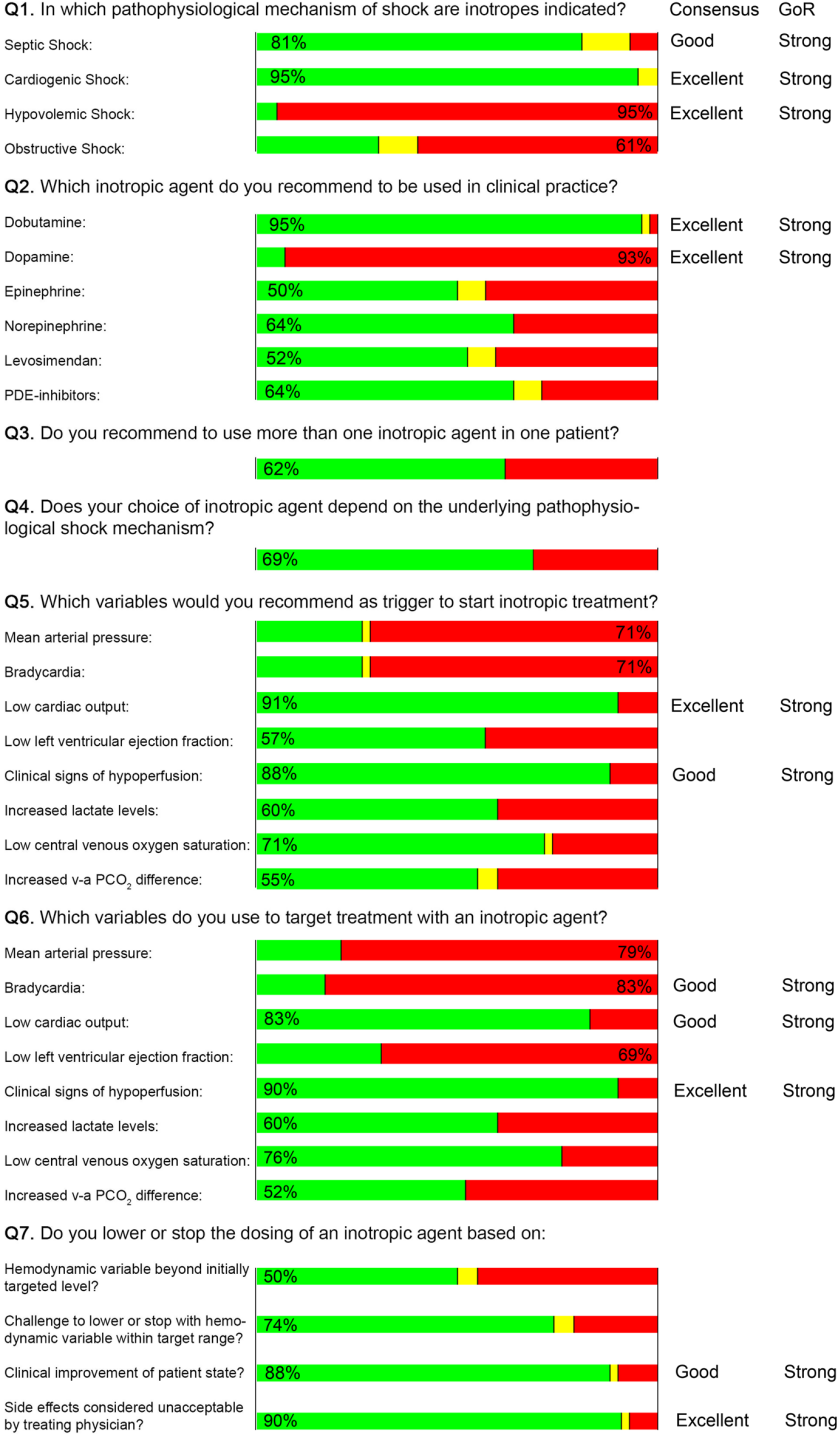
Expert answers to the first questionnaire and level of agreement. Answers are visualized as percentages. Positive answers are presented in green, conditional answers are presented in yellow, negative answers are presented in red. *PDE* phosphodiesterase, *v-a PCO*_*2*_ veno-arterial PCO_2_ difference, *GoR* grade of recommendation

Forty-two selected experts gave their recommendations for clinical use of inotropes by responding to the expert-opinion questionnaire (Fig. 1), and 40 of them replied to a follow-up questionnaire (Table 2).

**Table 2:** Second round questionnaire to 40 experts on inotrope use

Question	Answer
In a terminological sense (when discussing with fellow colleagues and/or researchers), do you refer to norepinephrine as a vasopressor?	
Exclusively a vasopressor	65%
It is context dependent and not a fixed term in discussions with fellow colleagues and/or researchers	35%
In your physiological understanding and your treatment approach, do you consider norepinephrine:	
Exclusively a vasopressor (i.e., you only use it to modify blood pressure)	42%
A vasopressor and an inotrope (i.e., in your treatment strategy, you consider it a drug to efficiently modify both blood pressure and cardiac output)	57%
What is your first-line inotrope?	
Dobutamine	82%
Epinephrine	5%
Levosimendan	2%
Milrinone	2%
Norepinephrine	8%
Do you recommend a PDE-inhibitor in right ventricular failure?	
Yes	65%
No	35%

Experts achieved excellent agreement (95%) on the statements that inotropes may be indicated in cardiogenic shock, that inotropes are not indicated in hypovolemic shock, that dobutamine but not dopamine can be used for treating circulatory shock in clinical practice, that a low CO can be used *as a trigger* for starting inotropic treatment, that clinical signs of hypoperfusion can be used *as a target* for inotropic treatment, and to lower or stop inotropic dosing, if patients experience unacceptable side effects. Other recommendations did not reach excellent agreement and for some the level of agreement was weak.

In general, experts individually stated that a recommended trigger for inotropic treatment should also be a target for the treatment (see Table 3). An exception was LVEF, where 14 of the 24 experts, who did use low LVEF as a trigger for inotropic treatment, did not consider LVEF as a target for the treatment, and three of the 18 experts, who did not choose low LVEF as a trigger did recommend using LVEF as a target for the treatment (Table 4).

**Table 3:** Summary of consensus among experts and the degree of recommendations

	Degree of consensus	Grade of recommendation
Inotrope indications		
1. Inotropes are indicated in septic shock	Good	Strong
2. Inotropes are indicated in cardiogenic shock	Excellent	Strong
3. Inotropes are NOT indicated in hypovolemic shock	Excellent	Strong
Choice of inotrope		
4. Dobutamine is the first-line inotrope	Good	Strong
5. Dopamine is NOT a recommended inotrope	Excellent	Strong
Triggers		
6. Low cardiac output is a trigger for inotropic treatment	Excellent	Strong
7. Signs of hypoperfusion are a trigger for inotropic treatment	Good	Strong
Targets		
8. Low cardiac output is a target for inotropic treatment	Good	Strong
9. Signs of hypoperfusion are a target for inotropic treatment	Excellent	Strong
Dosing should be lowered or stopped when		
10. Patients experience unacceptable side effects	Excellent	Strong
11. Patients show clinical improvement	Good	Strong

**Table 4:**
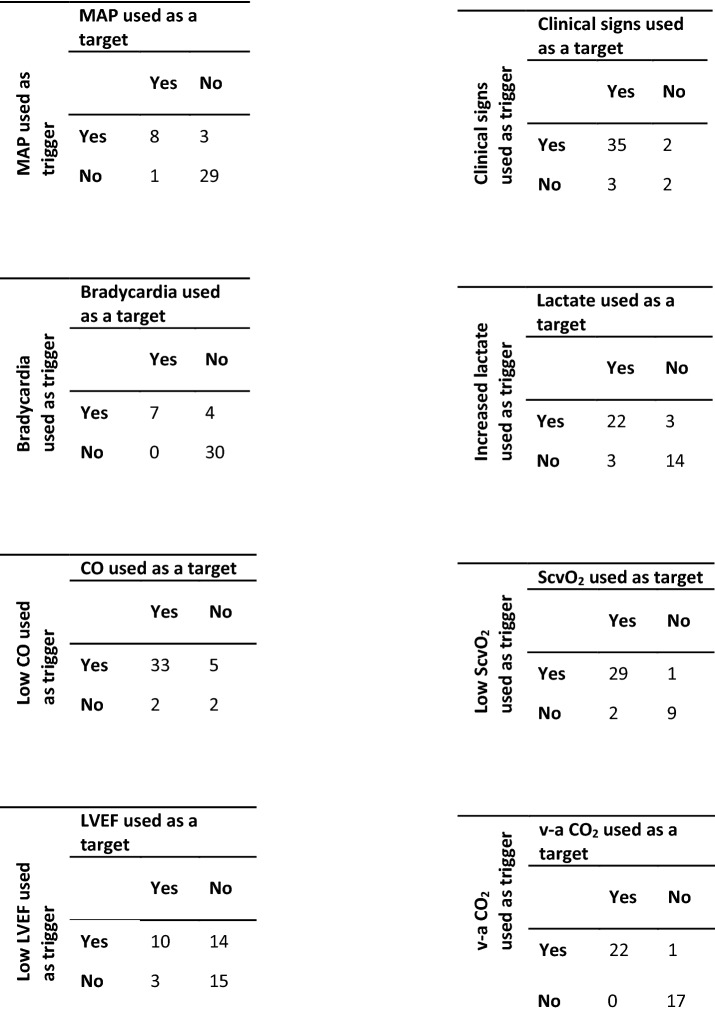
Answers from 42 experts on questions Q5 and Q6 from Fig. 1, where the view on *triggers* and *targets* for inotropic use are combined. Conditional answers for Q5 in triggers (marked yellow in Fig. 1) are not represented in this table. There was one conditional Q5 answer for MAP, Bradycardia, and ScvO_2_, and two for v-a CO_2_

## Discussion

According to the results of this international survey, preferences around the use of inotropes differ among physicians. Most physicians (84%) chose dobutamine as their first-line inotrope, and dopamine, levosimendan, and milrinone (or another PDE-inhibitor) were considered first-line in up to 5% of respondents for patients with circulatory shock. Furthermore, the reasons for using an inotropic agent were diverse. Also, the variation in the primary therapeutic target was diverse, where CO, ScvO_2_, lactate level and urine output were all well-represented answers among the respondents. Furthermore, the reasons for adding a vasopressor/inotrope if the patient did not respond to the inotropic agent administered to the patient were virtually uniformly distributed among the respondents, underscoring that balancing maximal doses, side effects, possible synergistic drug effects, etc., is challenging for clinicians in late/critical stages of circulatory shock.

The heterogeneous choices of physicians when it comes to inotropes may have various reasons. First, no solid evidence is available to support choosing one agent over another [12]. Recently, meta-analyses showed that for many inotropes evidence to support benefit is absent or weak [17, 18, 30]. Moreover, even a statistically significant effect should still be interpreted with caution since the effects might be small and the clinical relevance uncertain. Second, the evidence is sparse and not robust, not only because of between-trial heterogeneity, but also because of high within-trial patient heterogeneity, combined with little or no individualization in the treatment protocols. In turn, an effect of an inotrope might be present for certain (groups of) patients equalized by harm of the same inotrope in other (groups of) patients in the same trial [31]. As part of patient heterogeneity, the underlying pathophysiology and its impact on hemodynamics may be incompletely understood and therefore, choosing the right agent might be difficult. Third, the optimal therapeutic targets for individual patients or groups of patients are unknown. More data have recently become available supporting different targets in different patients, an example being blood pressure [32]. Furthermore, specific targets for a variable such as CO or cardiac index might be suboptimal. For one patient, a CO of 3.0 L min^−1^ might be sufficient to maintain organ perfusion, for another patient, this level of CO might be associated with organ hypoperfusion and organ dysfunction. Clearly, bedside titration of inotropes, based on individual patient responses, seems the most rational approach, but defining what those resuscitation targets should be, remains difficult. Finally, despite being available for many years (except for levosimendan in some countries), the optimal use of inotropes is incompletely understood, particularly beyond the choice of the first-line agent. Optimal treatment concepts for timing, dosing, interaction, and preferred combination of agents remain ill-defined.

Standard of care or daily practice is obviously not uniform among the respondents. Solid meta-analyses of all inotropes, performed according to contemporary standards and taking into account bias from funding sources, should become available and updated if new evidence arises [17, 30]. Outcome measures should be uniformly defined and incorporate patient-centred outcomes and not limited to surrogate outcomes such as CO. Therefore, triggers and goals/targets for treatment should be optimized by interpreting evidence of studies on hemodynamic monitoring.

Although primarily being a vasopressor, norepinephrine (in combination with dobutamine) was considered a preferred catecholamine for the treatment of circulatory shock. Even among experts there was disagreement on whether norepinephrine should be considered a pure vasoconstrictor or an agent with combined vasopressor and inotropic effects. Actually, through beta-1 adrenergic receptor stimulation, norepinephrine has been shown to increase systemic and microcirculatory blood flow along with blood pressure and preload in patients with septic shock [33–36]. Some clinicians might think of norepinephrine and also epinephrine as pure vasopressors because of their most dominant physiological effect, whereas others see it as a vasopressor with clinically relevant inotropic effects that may be enough to support contractility as a single agent. The difficult to target inotropic effect (these agents are titrated primarily based on their vasopressor effect) and their potential arrhythmogenic effects at high doses should be taken into account when these agents are used in this context. Epinephrine was hardly cited, possibly due to studies indicating safety concerns [37, 38].

Another interesting result is that most experts recommended using more than one inotropic agent in the same patient. Reasons for this might be synergistic effects by adding an independent mechanism of action, e.g., in case of adrenoceptor downregulation, or the wish to limit the dose and side effects of each agent [6].

The majority of the respondents of the survey as well as the experts chose dobutamine as preferred inotrope in patients with hypoperfusion to increase CO, which is in accordance with current guidelines [8, 9, 24]. More evidence might come from the ongoing ADAPT multicenter trial (ClinicalTrials.gov ID: NCT04166331), which tests the hypothesis that dobutamine will reduce tissue hypoperfusion and associated organ dysfunctions in patients with septic shock and associated septic cardiomyopathy.

Since clinicians prefer having recommendations accompanying evidence summaries in the context of low certainty of evidence [39], we asked international experts in the field to draft and agree on recommendations regarding inotropic treatment. In general, experts agreed that a recommended trigger for starting inotropic treatment should also be a therapeutic target, except for LVEF. Less than half of the experts using LVEF as a trigger for the use of an inotrope, also considered LVEF as the target for this use. This might be due to LVEF not being a continuously available variable, and its value is considered less reliable since it is mostly based on rough estimation of echocardiographic images (eyeballing) rather than exact measurements in clinical practice.

In view of the lack of evidence on the use of inotropes in circulatory shock, we suggest the following research agenda for the coming years:

1. Determine univocal and personalized triggers and targets to start inotrope therapy in circulatory shock states.
  Current evidence and expert opinion differ on the initial triggers to start and then guide inotrope therapy as targets in patients with circulatory shock. Consensus needs to be established on which triggers and target endpoints to use, ideally based on data rather than on expert opinion alone. These could be macro-hemodynamic values (e.g., cardiac output), surrogates of regional (organ) blood flow, or microcirculatory values (tissue perfusion, peripheral circulation) [40].
  As an example, interventional trials have used various triggers to initiate inotropic therapy such as “shock”, “low ejection fraction”, “low cardiac index” or “low SvO_2_ not responding to fluids”. Also, some trials use a fixed dose while others attempt to reach a given value of cardiac index or SvO_2_ or an improvement in a given variable (lactate, capillary refill time, microcirculation variables, etc.). It should be determined whether these triggers and targets should be identical for all patients or individualized based on cardiac function, organ perfusion, and underlying patient condition establishing an individualized benefit/risk profile.
2. Determine pharmacokinetics and pharmacodynamics of inotropes in shock.
  Little is known about the pharmacokinetics and pharmacodynamics (e.g., clearance) of available inotropes in the presence of shock. Information on clearance and uptake of inotropes in shock may have implications for specific aspects related to the timing of interventions, the weaning of these drugs, limiting the risk of delayed hemodynamic failure or rebound effects. This might also include research on the concomitant use of other medication (e.g., beta-blockers) and the effects of the various inotropes on the host (inflammatory or immune) response [41]. Finally, the individual variation in responses to inotropic drugs related to genetic related alterations in receptors and/or signaling pathways should be evaluated.
3. Compare and combine available inotropic agents and identify new, safer inotropes.
  Further multicenter randomized controlled trials (with adaptive designs) are needed to compare different inotropic agents (a vs. b) and their combinations (a + b vs. a or b alone) on different outcomes such as organ function, adverse effects and survival. For instance, combining two inotropic agents acting through different mechanisms or receptors (e.g., dobutamine + levosimendan) could permit minimizing the doses of each drug, thus reducing the incidence of adverse effects and increasing safety. The choice of combination should be based on the pharmacologic properties of the different agents (see point 2). New, non-catecholamine inotropic agents that are not associated with side effects such as arrhythmia or hypotension should be identified and tested.
4. Combine inotrope therapy with personalized care bundle.
  While inotrope use needs to be personalized in future research, the other mainstays of circulatory shock treatment must be employed in a similar personalized manner to improve comparability. For instance, optimal MAP targets in circulatory shock and the role of fluid therapy should ideally be established as these will influence inotrope therapy. However, this in itself will be challenging as there is no current consensus on types of fluid, monitoring and other interventions being delivered, nor on how to adopt an optimal personalized approach. For example, a one-size-fits-all approach for MAP targets cannot be optimal.
5. Develop and implement core outcome sets for patients with circulatory shock.
  Core outcome sets (i.e., standardized collection of outcomes measured and reported in all trials for a specific clinical area) should be developed for circulatory shock research due to established inconsistencies in trial outcome selection. Any new trial assessing the benefit/risk of inotropes should include the selection of an adequately targeted study population to improve the “noise/signal ratio” inherent to heterogeneous cohorts (in terms of hemodynamic profile).
6. Evaluate the impact of prolonged (> 72 h) inotropic therapy on myocardial energetics.
  Experimental and clinical data on inotrope use demonstrate direct effects of inotropes on myocardial injury, energetics and modulation of the immune/inflammatory response. The relevance of this to further organ injury and patient outcomes needs to be established. Data from experimental and clinical studies in chronic heart failure suggest that long-term inotropic therapy leads to interstitial calcinosis, myocardial fibrosis and contraction band necrosis [42]. Does this also apply to the context of shock where the duration of inotropic stimulation is expected to be shorter (less than one month)? What is the maximal duration of intravenous inotropic therapy before receptor down regulation, diastolic dysfunction, myocardial injury, and persistent arrhythmias develop in this setting?
7. Establish specific use of inotropes in patients under mechanical circulatory support.
  The use of inotropic agents should be adapted in patients under mechanical circulatory support for cardiogenic shock secondary to acute myocardial infarction. When are these agents indicated in this specific setting, and which hemodynamic targets should be used? The purpose of inotropic stimulation and the choice and doses of the inotropic agent may not be identical at the initiation of mechanical circulatory support, during the maintenance phase, or during the weaning process.
8. Evaluate the best hemodynamic strategy in predominant right ventricular failure.
  In patients with circulatory shock, which is predominantly associated with right ventricular failure, the question should be answered by comparative effectiveness trials if inotropic agents or vasopressors (e.g., norepinephrine to increase coronary perfusion pressure) should be preferred.
9. Better define the interaction between IV fluids and vasoactive agents.
  The physiologic interplay between vasoactive agents and intravenous fluids is evident, but the scientific evidence in terms of comparative effectiveness trials (fluids vs. early vasopressor use, addition of inotropes, etc.) is scarce. For instance, inotropic agents can only increase myocardial contractility, lusitropy, and heart rate. They do not primarily increase cardiac output. For cardiac output to increase there also needs to be sufficient blood volume and vascular tone, as known from the poor impact of inotropic agents in hemorrhagic shock and profound vasoplegia. Therefore, the optimal vasopressor/fluid/inotrope ratio remains to be determined at the individual level.

Ongoing and upcoming studies such as ADAPT (NCT04166331: Effects of dobutamine on tissue hypoperfusion and associated organ dysfunctions in patients with septic shock and associated septic cardiomyopathy) and LevoHeartShock (NCT04020263: Early use of levosimendan versus placebo on top of conventional inotropes in patients with cardiogenic shock) will probably provide important answers to some of these questions.

## Limitations

The number of responses is considered high (corresponding to 27% of ESICM members who opened the e-mail invite), but the methods used to invite individuals to respond did not allow us to report a conclusive response rate. Therefore, response bias might be present, in which case, external validity could be somewhat hampered.

The results presented in this manuscript come from an online survey. Online surveys have limitations like potential multiple responses by a single person. We did not use cookies or log-file/IP address analyses to prevent multiple responses. On the other hand, individual persons are unlikely to spend time answering a survey more than once. Another limitation is the multiple-choice character of our survey, limiting answers to those offered. In addition, studies published after the survey was performed [38, 43–46] might have altered the answers of the respondents. Nevertheless, after careful analysis of the results of those studies we believe that the experts’ recommendation would not have changed significantly. Furthermore, the recommendations of experts can only reach excellent agreement if the available evidence is solid and clear. The evidence for inotropes in circulatory shock lack this evidence for many questions raised. Furthermore, both patients and studies show high heterogeneity. Therefore, recommendations should be interpreted with caution.

## Conclusions

In conclusion, the use of inotropes in critically ill patients is quite heterogeneous as reported by individual caregivers. International experts recommend the use of inotropes in septic and cardiogenic shock (but not in hypovolemia), using an inadequate CO and signs of tissue hypoperfusion as triggers and targets for treatment, and adverse effects and clinical improvement as stopping/weaning criteria. While experts recommend using dobutamine as the first-line agent, they recommend against the use of dopamine. Future studies reporting patient-centred outcomes should focus on specific subpopulations based on prespecified and measurable triggers, targets, and with clear stopping criteria in order to ensure comparability across trials. This would allow a better summary of the evidence and its implementation in future guidelines.

## Supplementary Information

**Additional file 1: Table S1.** Baseline characteristics of survey respondents.

## Data Availability

The data of the survey are available from the corresponding author upon reasonable request.

## Abbreviations

ABP: Arterial blood pressure
CHERRIES: Checklist for Reporting Results of Internet E-Surveys
CO: Cardiac output
CVP: Central venous pressure
ESICM: European Society of Intensive Care Medicine
ICU: Intensive care unit
IC: Intensive care
LVEF: Left ventricular ejection fraction
MAP: Mean arterial pressure
NE: Norepinephrine
PDE: Phosphodiesterase
PAOP: Pulmonary artery occlusion pressure
SSC: Surviving Sepsis Campaign
ScvO_2_: Central venous oxygen saturation
v-a PCO_2_: Venous-to-arterial carbon dioxide pressure difference
