# Supplementary material for: Current use of inotropes in circulatory shock
Source: Ann Intensive Care. 2021 Jan 29;11:21. doi: 10.1186/s13613-021-00806-8 (PMC7846624; doi:10.1186/s13613-021-00806-8)
Supplement: Supplementary file 1 — Additional file 1: Table S1. Baseline characteristics of survey respondents. [file 13613_2021_806_MOESM1_ESM.docx]

**eTable 1.** Baseline characteristics of survey respondents

|  |  | Response rate | | | | | |
| --- | --- | --- | --- | --- | --- | --- | --- |
|  |  | Total | | Europe | | Outside Europe | |
| Valid respondents |  | 839 | (100%) | 546 | (65%) | 293 | (35%) |
| Main specialty area | | | | | | | |
|  | Intensive Care | 545 | (65%) | 313 | (57%) | 232 | (79%) |
|  | Anesthesiology | 197 | (23%) | 164 | (30%) | 33 | (11%) |
|  | Internal medicine | 53 | (6%) | 44 | (8%) | 9 | (3%) |
|  | Surgery | 8 | (1%) | 3 | (0.5%) | 5 | (2%) |
|  | Other | 36 | (4%) | 22 | (4%) | 14 | (5%) |
| Experience as intensivist | | | | | | | |
|  | Full time >5 years | 445 | (53%) | 282 | (52%) | 163 | (56%) |
|  | Full time 2-5 years | 98 | (12%) | 49 | (9%) | 49 | (17%) |
|  | Full time <2 years | 46 | (5%) | 26 | (5%) | 20 | (7%) |
|  | Part time intensivist | 141 | (17%) | 116 | (21%) | 25 | (9%) |
|  | Not specialized (yet) | 108 | (13%) | 73 | (13%) | 35 | (12%) |
| Type of institution | | | | | | | |
|  | University hospital | 353 | (42%) | 262 | (48%) | 91 | (31%) |
|  | Non-university public hospital | 183 | (22%) | 149 | (27%) | 34 | (12%) |
|  | University affiliated hospital | 178 | (21%) | 100 | (18%) | 78 | (27%) |
|  | Private hospital | 113 | (13%) | 31 | (6%) | 82 | (28%) |
|  | Other | 12 | (1%) | 4 | (1%) | 8 | (3%) |
| Type of ICU | | | | | | | |
|  | Mixed ICU | 627 | (75%) | 408 | (75%) | 219 | (75%) |
|  | Surgical ICU | 88 | (10%) | 68 | (12%) | 20 | (7%) |
|  | Medical ICU | 83 | (10%) | 50 | (9%) | 33 | (11%) |
|  | Other | 41 | (5%) | 20 | (4%) | 21 | (7%) |
| Number of ICU beds | | | | | | | |
|  | ≤5 | 23 | (3%) | 16 | (3%) | 7 | (2%) |
|  | 6-10 | 221 | (26%) | 176 | (32%) | 45 | (15%) |
|  | 11-15 | 188 | (22%) | 135 | (25%) | 53 | (18%) |
|  | 16-20 | 150 | (18%) | 89 | (16%) | 61 | (21%) |
|  | ≥20 | 257 | (31%) | 130 | (24%) | 127 | (43%) |
| Number of patients admitted per year | | | | | | | |
|  | <500 | 188 | (22%) | 135 | (25%) | 53 | (18%) |
|  | 500-1000 | 291 | (35%) | 193 | (35%) | 98 | (33%) |
|  | 1001-1500 | 178 | (21%) | 115 | (21%) | 63 | (22%) |
|  | 1501-2000 | 92 | (11%) | 58 | (11%) | 34 | (12%) |
|  | >2000 | 90 | (11%) | 45 | (8%) | 45 | (15%) |

With permission from Scheeren TWL et al. [6]. <https://eur03.safelinks.protection.outlook.com/?url=http%3A%2F%2Fcreativecommons.org%2Flicenses%2Fby%2F4.0%2F&amp;data=04%7C01%7Ct.w.l.scheeren%40umcg.nl%7C2921be92f3344a13853b08d89e781b15%7C335122f9d4f44d67a2fccd6dc20dde70%7C0%7C0%7C637433585968148566%7CUnknown%7CTWFpbGZsb3d8eyJWIjoiMC4wLjAwMDAiLCJQIjoiV2luMzIiLCJBTiI6Ik1haWwiLCJXVCI6Mn0%3D%7C1000&amp;sdata=x%2BFlJmHYU8La63GE%2FUao2oVUIsZ9CWxU9otaRjsYURw%3D&amp;reserved=0> , no changes made.
